# Optimization of *Plasmodium vivax* infection of colonized Amazonian *Anopheles darlingi*

**DOI:** 10.1038/s41598-023-44556-y

**Published:** 2023-10-24

**Authors:** Alice O. Andrade, Najara Akira C. Santos, Alessandra S. Bastos, José Daniel C. Pontual, Cristiane S. Araújo, Analice S. Lima, Leandro N. Martinez, Amália S. Ferreira, Anna Caroline C. Aguiar, Carolina B. G. Teles, Rafael V. C. Guido, Rosa A. Santana, Stefanie C. P. Lopes, Jansen F. Medeiros, Zaira Rizopoulos, Joseph M. Vinetz, Brice Campo, Marcus Vinicius G. Lacerda, Maisa S. Araújo

**Affiliations:** 1 Plataforma de Produção e Infecção de Vetores da Malária (PIVEM)/Laboratório de Entomologia, Fiocruz Rondônia, Porto Velho, Rondônia Brazil; 2 Programa de Pós-Graduação em Saúde Publica, Faculdade de Saúde Pública, Universidade de São Paulo, São Paulo, Brazil; 3 Programa de Pós-Graduação em Biologia Experimental, Fundação Universidade Federal de Rondônia, Porto Velho, Rondônia Brazil; 4 Programa de Pós-Graduação em Conservação e uso de Recursos Naturais – PPGReN, Fundação Universidade Federal de Rondônia, Porto Velho, Rondônia Brazil; 5 Faculdades Integradas Aparício Carvalho (FIMCA), Porto Velho, Rondônia Brazil; 6 Plataforma de Bioensaios de Malária e Leishmaniose da Fiocruz (PBML), Fiocruz Rondônia, Porto Velho, Rondônia Brazil; 7 Departamento de Biociência, Universidade Federal de São Paulo, Santos, São Paulo Brazil; 8 Rede de Biodiversidade e Biotecnologia da Amazônia Legal – BIONORTE, Porto Velho, Rondônia Brazil; 9 São Carlos Institute of Physics, University of Sao Paulo, São Carlos, São Paulo Brazil; 10 Instituto de Pesquisa Clínica Carlos Borborema, Fundação de Medicina Tropical Dr. Heitor Vieira Dourado, Manaus, Brazil; 11 Programa de Pós-Graduação em Medicina Tropical, Universidade do Estado do Amazonas, Manaus, Brazil; 12 Instituto Leônidas & Maria Deane, FIOCRUZ, Manaus, Brazil; 13 Medicines for Malaria Venture, Geneva, Switzerland; 14 Section of Infectious Diseases, Department of Internal Medicine, Yale School of Medicine, New Haven, CT USA; 15 Alexander von Humboldt Institute of Tropical Medicine and Faculty of Sciences, Universidad Peruana Cayetano Heredia, Lima, Peru

**Keywords:** Parasitology, Parasite biology

## Abstract

Obtaining *Plasmodium vivax* sporozoites is essential for in vitro culture of liver stage parasites, not only to understand fundamental aspects of parasite biology, but also for drug and vaccine development. A major impediment to establish high-throughput in vitro* P. vivax* liver stage assays for drug development is obtaining sufficient numbers of sporozoites. To do so, female anopheline mosquitoes have to be fed on blood from *P. vivax*-infected patients through an artificial membrane-feeding system, which in turns requires a well-established *Anopheles* colony. In this study we established conditions to provide a robust supply of *P. vivax* sporozoites. Adding a combination of serum replacement and antibiotics to the membrane-feeding protocol was found to best improve sporozoite production. A simple centrifugation method appears to be a possible tool for rapidly obtaining purified sporozoites with a minimal loss of yield. However, this method needs to be better defined since sporozoite viability and hepatocyte infection were not evaluated.

## Introduction

Malaria is caused by parasites of the genus *Plasmodium* spp., transmitted by transfer of sporozoites during blood feeding to humans by infected female *Anopheles* spp. mosquitoes^1^. The sporogonic cycle in the mosquito vector undergoes several bottlenecks, such as pre-fertilization events (gametocytes to zygotes), transition from ookinetes to oocysts (during midgut invasion) and invasion of the salivary glands by the sporozoites^2,3^. Consequently, not all gametocytes that are ingested by mosquitoes result in the presence of sporozoites in the salivary glands^4^.

*Plasmodium vivax* has a wider distribution than *P. falciparum*, and is the dominant malaria parasite in most countries outside of sub-Saharan Africa, especially in Southeast Asia and South America^5^. Treatment and control of vivax malaria is challenging because the parasite produces dormant stages, hypnozoites, in hepatocytes. This dormant stage can remain in the liver for years, reactivating and causing relapsing malaria infections at any time and maintaining the parasite in endemic settings^6^. The 8-aminoquinolones primaquine and tafenoquine are active against liver form; however, they are contraindicated in individuals with glucose-6-phosphate dehydrogenase (G6PD) deficiency, pregnant women and children below 6 months of age^7,8^ and require cellular metabolism to be transformed into their active forms. Hence for drug development against *P. vivax* liver forms, a scalable sporozoite-hepatocyte infection system is needed, which requires obtaining large numbers of sporozoites. Hence, to achieve malaria elimination and eradication, there is an urgent need to study the biology of *P. vivax* liver stage parasites, as well as testing anti-hypnozoite drug candidates^9,10^.

*P. vivax* liver stage research in South America remains scant^11^, probably due to the difficulty in performing in vitro* P. vivax* liver stage culture. A main obstacle in the development of high throughput in vitro *P. vivax* assays is the sustainable access to a large amount of viable sporozoites with high infectivity. As there is no long-term *P. vivax* blood stage culture, the only available way of obtaining *P. vivax* sporozoites is by obtaining infective gametocytes from infected humans, feeding to permissive mosquitoes (which has to be done in proximity to the parasite donor) and dissecting infected salivary glands for sporozoites. Therefore, the study of vivax malaria is primarily restricted to *P. vivax-*endemic areas.

The primary vector of malaria in the Amazonian basin, the main region of South American malaria transmission, is *Nyssorhynchus* (*Anopheles*) *darlingi* (*An. darlingi*). This anopheline species was first adapted for rearing under laboratory conditions fewer than ten years ago. Currently, there are four independent colonies of this species in the Amazon region^12–15^. As such, this has allowed the development of studies on transmission blocking and *P. vivax*-*An. darlingi* interactions based on direct membrane feeding assay (DMFA)^11,16–25^. However, the *An. darlingi* colonies maintained in Peru^12,13,17^ and Brazil^14,21^ have only obtained a mean yield of 6539–8141 (range 57–98,600) and 1840–4086 (range 80–37,800) sporozoites per mosquito, respectively, a substantially lower yield than from the Asian vectors, *An. stephensi* (22,562; range 275–73,000)^26^ and *An. cracens* (26,112; range 328–79,310)^27^. Therefore, a primary step for developing an in vitro culture system of exoerythrocytic stage parasites in this region would be to improve the *P. vivax* infection in an *An. darlingi* model to enhance yield of infective sporozoites.

A previous study on an *An. darlingi* colony from the Brazilian Amazon found that colonized mosquitoes maintained a high susceptibility to *P. vivax* infection after generation F25^21^. Herein, the aim was to further improve the *P. vivax* sporozoite production in an *An. darlingi* colony from the Brazilian Amazon. Some factors were considered that can naturally affect *Plasmodium* development in *Anopheles* mosquitoes, which are described mainly in Asian and African mosquito vectors^26,28–30^. Different purification methods were also tested to obtain sporozoites faster than salivary gland dissection without purification.

## Results

### The effect of serum replacement on *Plasmodium vivax* infections

**Figure 1:**
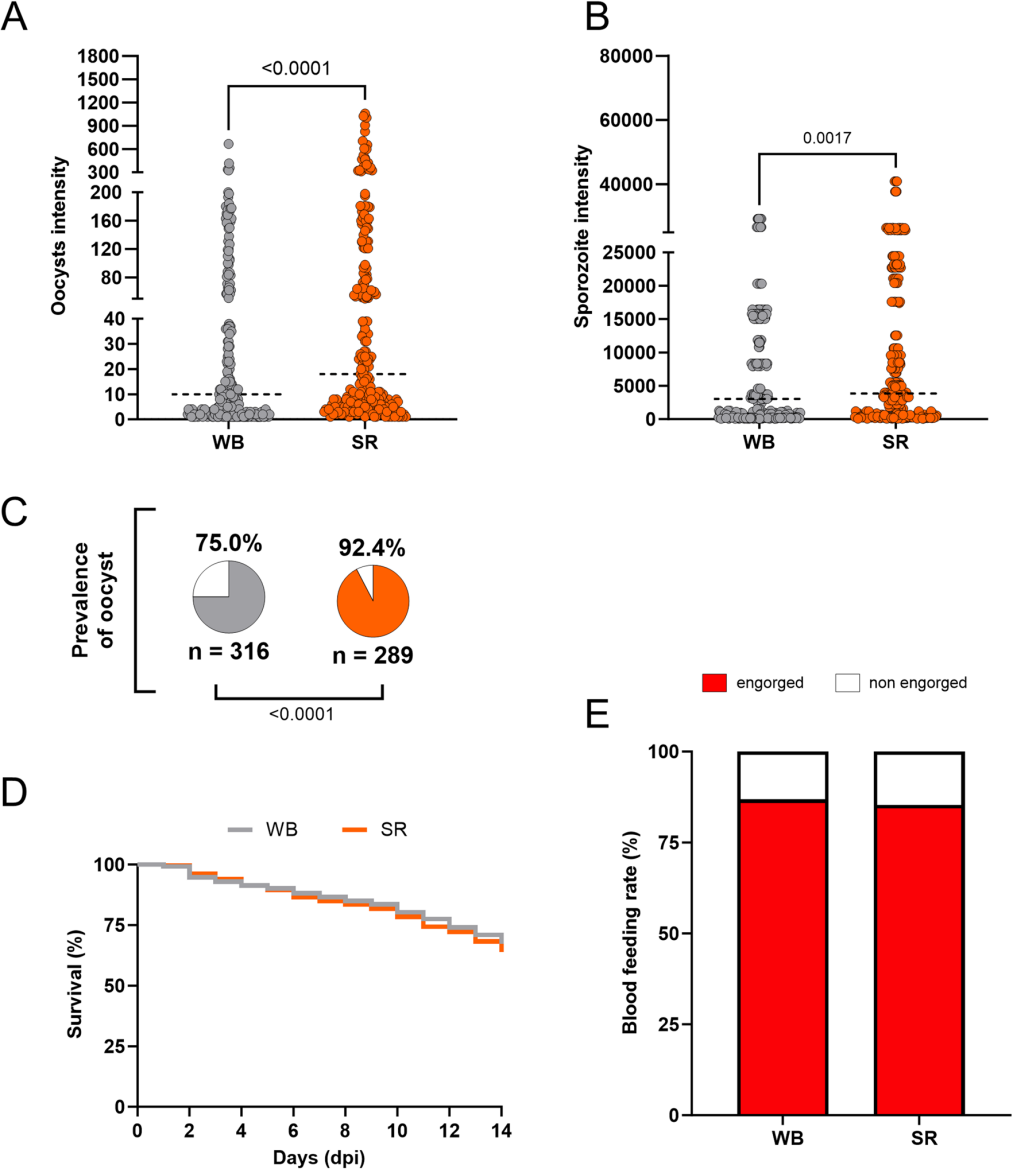
*Anopheles darlingi* infected with *Plasmodium vivax* blood samples with serum replacement. (**A, B**) *P. vivax* oocyst intensity and sporozoite intensity of *An. darlingi* fed on whole blood (WB) and fed on blood with serum replacement (SR). (**C**) Prevalence of infection in both experimental groups. *n* indicates the number of mosquitoes used in analyses. The dotted line indicates the median; gray = WB and orange = SR. (**D**) Survival curve of mosquitoes fed on whole blood (WB) and fed on blood with serum replacement (SR). (**E**) Blood feeding rate of *An. darlingi* fed on whole blood (WB) and blood with serum replacement (SR).

There is a concern that patient plasma in an endemic setting may have transmission-blocking factors that interfere with the infectivity of gametocytes in mosquitoes^4,17,26,31^. For this reason, the effect of serum replacement on *P. vivax* infections was assessed using *An. darlingi* from a well-established colony in Brazil. Ten independent feeding experiments with different *P. vivax* patient isolates were conducted (see Supplementary Data Table S1). Mosquitoes fed on serum replaced (SR) samples had a significantly higher prevalence (92.4%, *p* < 0.0001) (Fig. 1C), and infection intensity (median 18 oocysts/mosquito; U = 25,115; *p* < 0.0001, and a median of 3840 sporozoites/mosquito; U = 36,024; *p* = 0.0017) than those fed with whole blood (WB) (prevalence: 75.0%; median 10 oocysts/mosquito; 3040 sporozoites/mosquito) (Fig. 1A, B). Table 1 presents detailed data from the feeding experiments.

**Table 1:** Laboratory-colonized *Anopheles darlingi* with Brazilian *Plasmodium vivax* infected blood.

	Isolates	Gametocytes/µL	Blood feeding rate (%)	CI 95% of blood feeding rate	*p* value	Prevalence (%)	CI 95% of prevalence	*p* value	Intensity			*p* value
									Oocyst	*p* value	Sporozoite	
Whole blood (WB)	n = 10	105 (0–990)	86.8 (872/1004)	84.6–88.8	0.3154	75.0 (237/316)	69.9–79.4	< 0.0001	10 (1–667)	< 0.0001	3040 (80–29,200)	0.0017
Serum replacement (RS)			85.3 (853/1000)	82.9–87.3		92.4 (267/289)	88.6–94.9		18 (1–1059)		3840 (80–40,900)	
Control (WB)	n = 15	180 (30–1500)	84.6 (1191/1407)	82.6–86.4	–	83.4 (262/314)	78.9–87.1	–	67 (1–706)	–	9440 (80–79,040)	–
PABA			83.3 (1169/1403)	81.2–85.1	0.3376	85.5 (271/317)	81.1–88.9	0.4773	60 (1–604)	> 0.9999	4880 (80–71,360)	< 0.0001
PABA/PSG			82.7 (1164/1406)	80.7–84.6	0.1815	85.2 (265/311)	80.8–88.7	0.5430	67 (1–645)	> 0.9999	4960 (80–42,880)	< 0.0001
PABA/SR			78.1 (1097/1404)	75.9–80.2	< 0.0001	95.0 (287/302)	91.9–97	< 0.0001	97 (1–759)	0.0004	8400 (160–68,960)	0.3217
PABA/PSG/SR			75.6 (1063/1405)	73.3–77.8	< 0.0001	92.1 (281/305)	88.5–94.7	0.0010	112 (1–846)	< 0.0001	8333 (133–74,320)	0.0802
PSG/SR			85.2 (1193/1400)	83.2–86.9	0.6751	91.7 (286/312)	88.0–94.2	0.0018	112 (1–1275)	< 0.0001	12,800. (1280–143,360)	< 0.0001

Gametocytes/µL, oocysts per mosquito and sporozoites per mosquito are expressed as median (max–min).

PABA = mosquitoes treated with 0.05% para-aminobenzoic acid; PSG = mosquitoes treated with 10 U/mL–μg/mL Pen-Strep and 15 μg/mL of gentamicin.

Excessive mortality rates were not registered between the groups (Fig. 1D). Furthermore, the blood-feeding rate was similar in the groups fed on WB (86.8%) and SR (85.3%) (*p* = 0.3154) (Fig. 1E).

### The effect of combination para-aminobenzoic acid (PABA), antibiotics, and serum replaced on *Plasmodium vivax* infections of *Anopheles darlingi*

**Figure 2:**
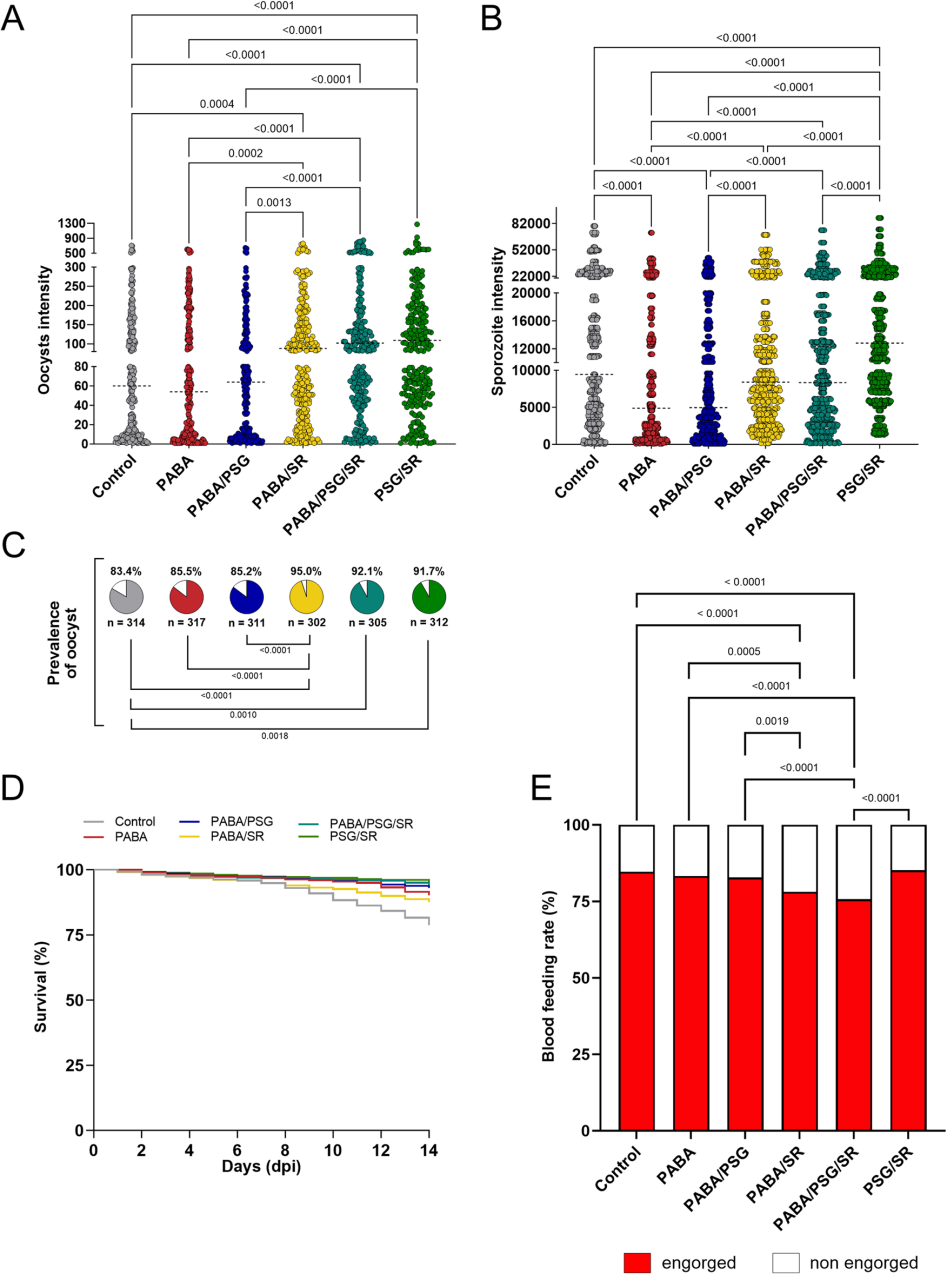
*Anopheles darlingi* under different combinations of para-aminobenzoic acid (PABA) supplementation, antibiotic treatment and serum replacement. (**A**) *Plasmodium vivax* oocyst and (**B**) sporozoite intensities of infection in *An. darlingi* under different combinations of treatments. (**C**) Prevalence of infection of mosquitoes under different treatments. Dotted lines indicate the median; *n* = indicates the number of biologically independent mosquito samples; gray = control, red = PABA, blue = PABA/PSG = , yellow = PABA/SR, dark green = PABA/PSG/SR, and light green = PSG/SR. (**D**) Survival curve of mosquitoes under different combinations of PABA, antibiotics (PSG) and serum replacement. (**E**) Blood-feeding rate of mosquitoes under different combinations of PABA, antibiotics (PSG) and serum replacement

Considering that other factors beyond immune molecules present in human plasma may influence *P. vivax* infection, such as the anopheline immune response to infection and the microbiota in the midgut and salivary glands^28^, we conducted different combinations of interventions. These interventions included para-aminobenzoic acid (PABA) supplementation, antibiotic treatment (Pen-Strep and gentamicin, identified as PSG) to eliminate or reduce the microbiota, and serum replacement in order to assess improvements in infection intensity were performed. A total of fifteen independent feeding experiments with different *P. vivax* patient isolates were carried out (See Supplementary Data Table S2). The prevalence of infection and oocyst intensity observed in mosquitoes treated with PABA or PABA plus PSG, and fed on WB, did not differ from each other or from the control group without any treatment (Fig. 2A–C, Table 1). However, a higher infection prevalence and oocyst intensity were observed in the groups that were fed with blood contain serum replacement, regardless of the treatment performed on the mosquitoes before the DMFA (Fig. 2A, C, Table 1).

Regarding sporozoite intensity, the results showed a different pattern. The control group exhibited a higher median of sporozoites per mosquito than the PABA and PABA/PSG groups (9440; 4880; *p* < 0.0001 and 4960; *p* < 0.0001, respectively) (Fig. 2B, Table 1). On the other hand, no significant difference in sporozoite intensity was found between the groups with different treatment protocols that were fed on SR blood or with the control group. However, the PSG/SR group demonstrated the highest sporozoites production per mosquito, with a median of 12,800 sporozoites/mosquito (*p* < 0.0001) (Fig. 2B, Table 1). These results suggest that mosquitoes supplementation with PABA does not increase the prevalence and intensity of oocysts and may even inhibit sporozoites production of their invasion of salivary glands.

Concerning the survival rate, a slight increase was observed in all treated groups compared to the control group (*p* < 0.0001). In general, all groups that were treated with PSG showed the highest survival rates (Fig. 2D, Table 2). Additionally, when the PABA treatment was combined with SR (PABA/SR) or both SR and PSG (PABA/PSG/SR), a significant decrease in the blood feeding rates was observed compared to the control group (PABA/SR = 78.1%; PABA/PSG/SR = 75.6; control = 84.6, *p* < 0.0001) (Fig. 2E). There was no significant difference among the control, PABA (83.3%, *p* = 0.3376) and PSG/SR (85.2, *p* = 0.6751) groups (Fig. 2E).

**Table 2:** Survival rate of laboratory-colonized *Anopheles darlingi* fed with Brazilian *Plasmodium vivax-*infected blood.

	Survival (%)	95% CI	*p* value
Whole blood (WB)	67.2	63.6–71.2	0.292
Serum replacement (SR)	63.9	60.2–67.9	
Control (WB)	78.8	76.3–81.3	-
PABA	90.1	88.3–92.0	0.0001
PABA/PSG	92.9	91.4–94.5	0.0001
PABA/SR	87.6	85.6–89.8	0.0001
PABA/PSG/SR	93.9	92.4–95.5	0.0001
PSG/SR	95.2	93.9–96.4	0.0001

### Improving the purification of sporozoites

**Figure 3:**
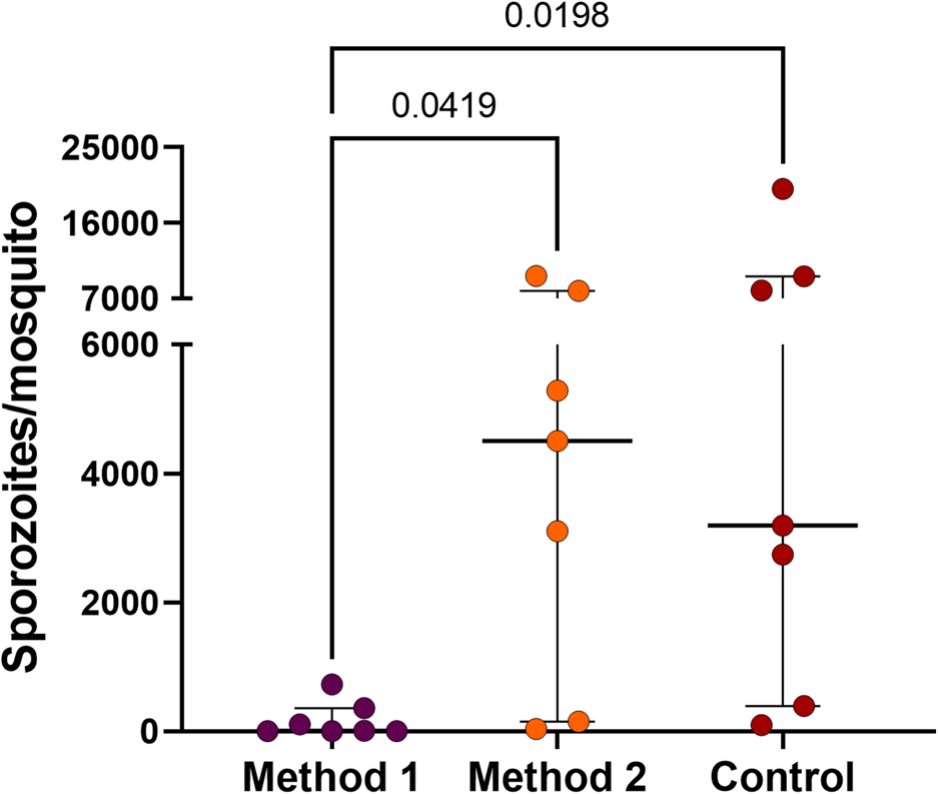
*Plasmodium vivax* sporozoite purification methods. The graph shows the amount of *P. vivax* sporozoites obtained in each extraction method experiment from *An. darlingi*. Each dot represents an independent feeding experiment. Error bars indicate interquartile range. Method 1: purification method used by Kennedy et al.^32^ and method 2: the method described by Ozaki et al.^33^.

An essential aspect in developing the liver stage assay is ensuring the viability and purity of the sporozoite fraction to maximize hepatocyte infectivity and minimize culture contamination. Moreover, it is crucial to have a procedure that reduces the time labor requirements, faciliting the development of high-throughput assays. Therefore, two sporozoite purification methods were tested in conjunction with the salivary dissection (control). These methods were previsouly described by Kennedy et al.^32^ (referred as “method 1”) and in Ozaki et al.^33^ (referred as “method 2”), and compared to salivary gland dissection without purification, which was considered as the control. A total of seven independent feeding experiments using different *P. vivax* patient isolates were conducted for this assessement (See Supplementary Data Table S3). Our data demonstrated that the method utilizing density gradient purification (method 1) resulted in a loss of over 90% (*p* = 0.0419) of the sporozoites when compared to the control group (median of sporozoites per mosquito = 9.7 and 3195.4, respectively). In contrast, method 2 showed a similar median of sporozoites per mosquito (4505.9 sporozoites per mosquito; *p* = 0.0198) compared to the control group (without purification) (Fig. 3). However, it is noteworthy that method 2 yielded a sporozoite fraction with greater contamination (bacteria and fungi) than the other two methods.

## Discussion

Here we report improvement of the yield of viable, infectious *P. vivax* sporozoites by modifying critical parameters of the membrane feeding and mosquito husbandry procedure. This is important for facilitating the development of new interventions such as new drugs and vaccines against *P. vivax.*

Implementation of in vitro pre-erythrocytic (PE) models for *P. vivax* in malaria endemic areas in Brazil requires the improvement of the experimental infection of the main local vector to standardize the DMFA and, thereby, produce more sporozoites. One of the limitations in the development of high-throughput in vitro* P. vivax* liver stage assays is sustainable access to a large number of viable sporozoites with high infectivity^34^. Since the establishment of *An. darling* colony in Brazil^14^, artificial infection has shown the susceptibility of this colony to local *P. vivax* isolates^18–22^. However, the infection intensity of the *An. darlingi* colony^12–14,17,18,21^ that has been obtained is below the ones described for Asian vectors, such as *An. stephensi*^26^ *An. cracens*^27^, and *An. dirus*^35^.

Different approaches were carried out to optimize *P. vivax* infection in the Brazilian *An. darlingi* colony. Corroborating with the findings from *An. stephensi*^26^, *An. dirus*^30^ and the Peruvian *An. darlingi* colony^17^. It became evident that immune factors present in the plasma of the *P. vivax* samples interfered with the Brazilian *An. darlingi* infection. A similar pattern has already been observed in *P. falciparum* samples using an *An. gambiae* colony^4,31^. The conditions in mosquito midgut stimulate the transition from gametocytes to gametes, which can be exposed to antibodies in the blood meal plasma, potentially reducing or preventing fertilization. The midgut environment may also stimulate white blood cell (WBC) mediated phagocytosis and the killing of the parasite during sexual stages^4,31^. Vallejo et al.^29^ described a negative correlation between the levels of IFN-γ, IL-10 and TNF in serum and Pvs25 expression in *P. vivax* blood samples from endemic areas of Colombia, indicating that the immune response may have an impact gametocytes maturation in the blood meal.

Besides the human immune factors present in plasma, mosquito diet supplementation can influence *Anopheles* susceptibility to *Plasmodium*^28,36^. For example, an increase in rodent malaria parasite infection was observerd in *An. stephensi* and *An. albimanus* by adding a metabolite, such as PABA, to the sugar solution prior to the infectious blood meal^37,38^. Although 0.05% PABA is included in the sugar solution in *P. vivax* infection using the Asian vector for sporozoite production^39–41^, our experiments provided no evidence that PABA enhances sporogonic development of *P. vivax* in *An. darlingi*. Similar results were reported by Beier et al.^28^ regarding *P. falciparum* development of in *An. gambiae* and *An. stephensi*. Although PABA is a metabolite essential for malaria parasite needs for de novo folate synthesis^42,43^, but its effect on the development of human malaria parasites in anopheline mosquitoes appears to differ from its action on parasite stages in the vertebrate host.

The mosquito midgut microbiota has the potential for reducing *Plasmodium* infectivity to mosquitos via the DMFA and is another parameter potentially to modify. The reduction or elimination of microbiota from African and Asian vectors using antibiotic treatment in their sugar solution increased *Plasmodium* infectivity^44–47^. Microbiota may influence mosquito immunity ^44^; or directly through metabolites action ^48,49^. Although our previous experiments showed no effect of *An. darlingi* microbiota on *P. vivax*^22^ as well as described in Peruvian *An. darlingi* colony^17^, our new data showed that the elimination of immune factors present in the plasma of *P. vivax* samples and adding antibiotics to the blood meal to reduce *An. darlingi* microbiota showed a significant increase in sporozoite intensity (increase in the median of sporozoites per mosquito 9440 (control) to 12,800(PSG/SR)). Converting this value to the mean to compare the mean number of sporozoites per mosquito, the PSG/SR group produced close to 20,000 sporozoites per mosquito (mean = 18,915 ± SEM 758.4), a mean that is similar to the mean registered for *An. stephensi* (~ 22,000 sporozoites per mosquito)^26^. We previously observed that the reduction in bacterial microbiota by treating the mosquitoes with antibiotics increased mosquito survival when compared with untreated mosquitoes^21^ in our Peruvian *An. darlingi* colony^17^. Similarly, our analyses showed an increase in survival in all groups treated with antibiotics, regardless of the combination. Additionally, the comparison between mosquitoes that fed on WB and SR showed no difference in survival and blood feeding rates. However, PABA treatment negatively affected the blood-feeding rate only when combined with SR and SR plus PSG treatment. Beier et al.^28^ previous described that PABA alone did not affect the survival and blood meals. The reason for the effect of PABA on blood-feeding only in combined with SR and SR plus PSG treatment is currently unknown.

Therefore, our results demonstrated the feasibility of increasing *P. vivax* infection and sporozoite levels in *An. darlingi* in mosquito feeding experiments, while maintaining the blood-feeding rate and improving the survival of sporozoite-infected mosquitoes.

The next crucial step was to standardize a protocol for obtaining a large number of sporozoites with low contamination levels. Traditional salivary gland dissection is labor-intensive and requires skilled personnel to ensure the integrity of salivary glands and prevent contamination, which can interfere with the in vitro hepatic stage cultivation. Method 1, which uses the Accudenz-based density gradient purification method; this purification technique was fast and effective for purifying malaria sporozoites by significantly reducing the contaminating mosquito debris and microbial burden associated with sporozoite isolation^32^. However, our attempts to use this method with *P. vivax* sporozoites from *An. darlingi* did not yield similar results as found in Orjuela-Sanchez et al.^11^ and Kennedy et al.^32^. Numerous sporozoites were lost compared to method 2^33^. The main difficulty was the identification of the bilayer interface, which was not readily visible. This difficulty arose because we followed the salivary gland dissection method described by Orjuela-Sanchez et al.^11^ rather than the original microdissection protocol recommended by Kennedy et al.^32^. Consequently, mosquito debris pellets at the bottom of the gradient and mosquito lipids floating at the top were also not visible. Further tests will be necessary to determine whether method 1 can be successfully adapted for *P. vivax* sporozoite purification from *An. darlingi*. On the other hand, method 2 showed the same number of sporozoites obtained using our control, the salivary gland dissection. Method 2 is based on a simple method for the rapid separation of sporozoites from infected mosquitoes using 1.5 mL tubes and speed centrifugation^33^. However, bacterial and yeast contaminants were found in the sporozoite samples of method 2. Hepatocyte infection and liver stage assays require sporozoite samples to be as clean as possible to avoid contamination of the hepatocyte cultures after infection. Therefore, the use of method 2 to purify sporozoites may not be appropriate for this goal and additional experiments are needed to evaluate, this method, including infection of hepatocytes with the obtained samples.

In conclusion, treatment of *An. darlingi* with antibiotics and an infective meal containing *P. vivax* with serum-replaced blood seems to be the best approach for obtaining a greater amount of live mosquitoes at day 14 post-feeding and *P. vivax* sporozoites in *An. darlingi*. Therefore, it is now possible to progress to the validation of sporozoite infectivity in an in vitro hepatocyte culture.

## Methods.

### Ethical approval

The protocol for collection blood from patients received approved from the Centro de Pesquisa em Medicina Tropical (CEPEM) Ethical Committee under protocol #28176720.9.0000.0011. Informed consent was obtained from all volunteers, and all procedures were performed in accordance with relevant guidelines and regulations.

### *Plasmodium vivax* gametocyte samples

*P. vivax* field isolates were obtained from individuals over 18 years of age who had been diagnosed with a mono-infection of vivax malaria at the CEPEM malaria clinic using microscopy. Ten milliliters of venous blood were collected from each volunteer in a heparinized tube.

After sample collection, all patients received treatment in accordance with the Brazilian Malaria Treatment Guidelines. Thick blood smears were prepared in duplicate using 10 μL of whole blood to quantify parasitemia via microscopy. *P. vivax* parasitemia and gametocyte counts were analyzed according to the WHO^50^. The remaining of the sample was stored at 37 °C and used in the DMFA.

### Mosquito rearing

A colony of *An. darlingi* has been maintained in the insectary of the Malaria Vector Production and Infection Platform (PIVEM), located in the Entomology Lab at Fiocruz Rondônia since 2018^14^. Mosquitoes were reared at temperature of 26 °C ± 1 °C with a relative humidity of 70% ± 10%, and a 12-h day/night cycle. Adults mosquitoes were provided with a 15% honey solution ad libitum, and 3–5-day-old female mosquitoes were selected for use in the DMFA.

### Direct membrane feeding assay with *Plasmodium vivax*

Mosquitoes were starved of the honey solution overnight before the day of infection, and approximately 100 mosquitoes were used in each cage. For each feeding session, between 1 and 2 mL of *P. vivax* infected whole blood or reconstituted blood (depending on the specific experiment) was added to a glass membrane feeder fitted with a parafilm membrane, and the temperature was maintained at 37 °C throughout the entire procedure^51^. The mosquitoes were allowed to feed on the infected blood for a period of 30 min. Subsequently, unfed or partially fed mosquitoes were removed, and the percentage of mosquitoes fully engorged mosquitoes was determined and recorded as the blood-feeding rate. The remaining mosquitoes were kept in the insectary under the same aforementioned conditions for 7 to 14 days before dissection. Cotton pads soaked in a 15% honey solution were provided immediately following blood-feeding and were replaced daily until the mosquitoes were dissected to assess malarial infection.

### Protocol for increasing *Plasmodium vivax* infection

#### Serum replacement of blood samples

The initial series of experiments aimed at optimizing the *An. darlingi* infection evaluated whether the host’s immune components present in the plasma influenced mosquito infection. To achieve this, each blood sample was divided into two equal volumes. One aliquot was used as whole blood (identified as WB), while the second aliquot was prepared for serum replacement (identified as SR). For the SR group, the blood was centrifuged at 1500 rpm for 10 min, resulting in the removal of plasma, which the replaced with an equivalent volume of heat-inactivated AB^+^ serum from healthy donors (30 min at 56 °C).

### Treatment of mosquitoes with different combinations of para-aminobenzoic acid (PABA) and antibiotic and serum replacement

**Figure 4:**
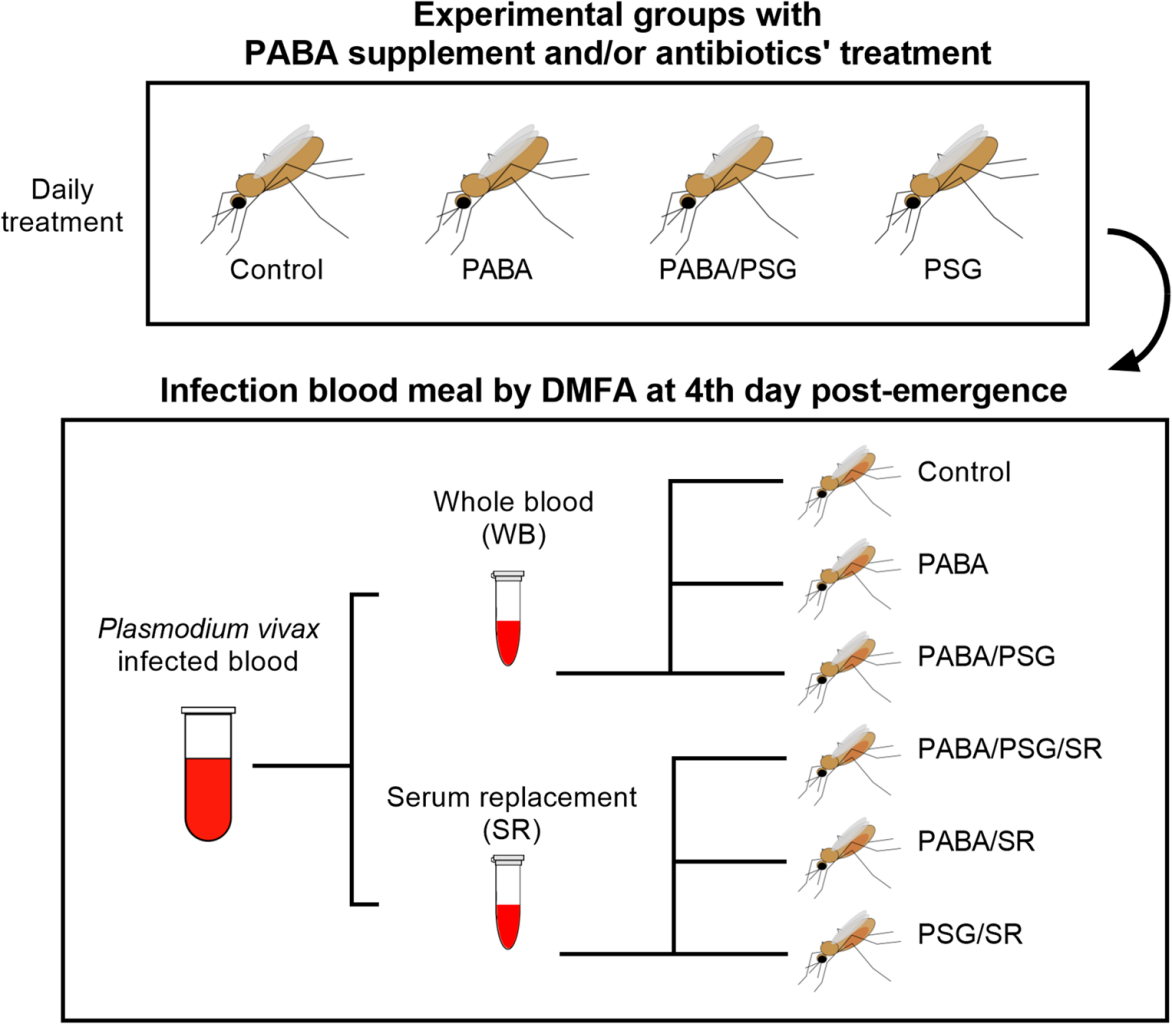
Scheme of mosquito treatment for DMFA with *Plasmodium vivax*-infected blood. Batches of approximately 100 *An. darlingi* were treated with a combination of para-aminobenzoic acid (PABA) supplement and/or antibiotics (PSG), daily after adult’s emergence and until day 14 post-feeding. *Plasmodium vivax* infected blood was offered via a DMFA to mosquito batches on the 4th day post emergence. Infected blood was divided in two aliquots. The first was offered to mosquitoes as whole blood (WB); and the second was prepared for serum replacement (SR) before being offered to the batches of the mosquitoes. Figure was generated using the following software: Power Point of Microsoft Office Package, Inkscape 1.3 (0e150ed6c4, 2023-07-21, dated July 2023) and Prism 10 for Windows 64-bit (Version10.0.2 Build 232).

The second set of experiments aimed to optimize the *An. darlingi* infection involved various combinations of mosquitoes treatment with the PABA supplemention and/or antibiotic treatment to assess their impact on mosquito infection. At the same time, some experimental groups had the infected blood sample prepared with or without serum replacement. Six distintic experimental groups were evaluated, each one containing approximately 100 mosquitoes per patient sample. These groups are identified as follows: (i) Control: mosquitoes without any treatment and fed with whole infected blood; (ii) PABA: mosquitoes receiving a 0.05% PABA supplement and fed with whole infected blood; (iii) PABA/PSG: mosquitoes that received a 0.05% PABA supplement plus antibiotics and fed with whole infected blood; (iv) PABA/PSG/SR: mosquitoes that received a 0.05% PABA supplement, plus antibiotics and fed with reconstituted blood; (v) PABA/SR: mosquitoes that received a supplement with 0.05% PABA and fed with reconstituted blood; and vi) PSG/SR: mosquitoes that received antibiotic treatment and fed with reconstituted blood (Fig. 4).

The PABA supplement and antibiotic treatment were initiated immediately after the adults mosquitoes emerged and continued until the mosquitoes were dissected. A 0.05% PABA solution was prepared by diluting the acid in a 15% aqueous honey solution. The antibiotic treatment consisted of a mixture containing 10 U/mL-10 μg/mL penicillin–streptomycin (Gibco™, catalog number: 15070063) and 15 μg/mL gentamicin sulfate (NovaFarma) in 15% aqueous honey solution.

### Mosquito dissection and parasite count

The oocysts in the midguts and sporozoites in the salivary glands were examined and counted using microscopy on the day 7 and 14 post-feeding, respectively. Each mosquito midgut was stained with 0.02% mercurochrome stain, and the number of oocysts per midgut was counted using a light microscope at 100 × magnification. For each sporozoite-positive mosquito batch, salivary glands from five mosquitoes were dissected and pooled in a microcentrifuge tube containing 15 μL of sterile serum-free RPMI1640 medium (Gibco™, catalog number: 61870–036), then ground with a sterile glass pestle. The released sporozoites were then counted using a Neubauer chamber to calculate the average number of sporozoite per mosquito.

### Blood-feeding rate and mosquito survival rate

The blood-feeding rate was appropriately recorded. The engorged mosquitoes were kept in plastic cups under conditions described above. Cotton pads soaked in a 15% honey solution were provided immediately after blood-feeding and changed daily until dissecting in order to assess malaria infection. The mosquito survival rate was calculated by comparing the number of viable mosquitoes on day 7 and 14 post-feeding with the initial number of engorged mosquitoes on day 0.

### *Plasmodium vivax* sporozoite purification

Considering the short lifespan and viability of sporozoites outside the mosquito salivary glands, it is important to rapidly obtain then use *P. vivax* sporozoites in procedures such as hepatocyte invasion assays. The conventional method for obtaining sporozoites involving dissecting the entire salivary glands with tweezers using a stereomicroscope. This procedure is a delicate, labor-intensive, and requires well-trained staff. Therefore, herein, two simple sporozoite purification methods, which have been previously described, were evaluated and compared to the traditional dissection method.

For this purpose, for each *P. vivax* isolate (n = 7), three cages of approximately 200 mosquitoes were submitted to DMFA using the serum replacement protocol described above. A batch of 10 mosquito midguts was dissected on the day 7 post-feeding to confirm the success of the infection. The experiment was continuated only if 10–100 oocysts per midgut were found in the batch of infected mosquitoes. If the infection met or exceeded threshold of 10–100 oocysts, then the remaining mosquitoes were kept in the insectary for acquisition of sporozoites. On day 14 post-feeding, one group of each feeding experiment was submitted to traditional salivary gland dissection, the second group was submitted to the sporozoite scalable density gradient purification method described by Kennedy et al.^32^ and adapted by Orjuela-Sanchez et al.^11^, and the last group was submitted to the method described by Ozaki et al.^33^. In this method, a pool of the first thorax portion of mosquitoes was loaded into a 0.1 mL tube with glass wool to serve as a filter within a 1.5 mL tube and underwent low-speed centrifugation for the separation of sporozoites. The traditional salivary gland dissection is identified here as the “control”, the sporozoite scalable density gradient purification method is identified as “method 1”^11,32^ and the method described by Ozaki et al.^33^ is identified as “method 2”. For each method, 10 µl of sporozoite solution was used to determine the number of sporozoites per mosquito using a Neubauer chamber as described above.

### Statistical analysis

Statistical analyses were carried out using GraphPad v.9.0 for MacOSX (GraphPad). For infections, differences in prevalence and blood-feeding rate were analyzed using the Chi-squared test. The blood-feeding rate was determined by proportion of full engorged mosquitoes after 30 min to blood feeding and infection prevalence was determined by proportion of mosquitoes infected with oocysts at 7 dpi. In the serum replacement experiment, differences in the median oocysts and the sporozoite burden between groups (infection intensity) were analyzed using a Mann–Whitney mean ranks test. For multiple comparisons, differences in prevalence and blood feeding rate were determined using a pairwise Chi-squared test, corrected multiple comparisons (Bonferroni), and the confidence interval was calculated using the Wald method. For multiple comparisons of infection intensity, the Kruskal–Wallis test followed by a post hoc Dunn’s test was applied. Kaplan Meier curves were used to estimate the survival rate of all experimental groups and *p* values were corrected for multiple comparisons (Bonferroni). To determine the best sporozoite purification method, the Kruskal–Wallis and post hoc Dunn’s test were used. All collected data are presented in tables and figures and supplementary data. If relevant (i.e., for experiments involving live vertebrates and/or higher vertebrates or human subjects/tissue samples), appropriate licensing committee approvals, any relevant details, confirmation of experimental performance in accordance with relevant guidelines and regulations, and informed consent (where applicable) must be included.

### Supplementary Information

Supplementary Information 1.

## Data Availability

The data for the current study are available from the corresponding author on reasonable request.
